# Rare diseases in China: analysis of 2014–2015 hospitalization summary reports for 281 rare diseases from 96 tertiary hospitals

**DOI:** 10.1186/s13023-019-1137-y

**Published:** 2019-07-01

**Authors:** Xinmiao Shi, Hui Liu, Siyan Zhan, Zhaoxia Wang, Lin Wang, Chongya Dong, Yanfang Wang, Chen Yao, Jie Ding, Yan Li

**Affiliations:** 1 Department of Pediatrics, Peking University First Hospital, Beijing, China; 2 Medical Informatics Center, Peking University, Beijing, China; 3 Department of Epidemiology and Bio-statistics, School of Public Health, Peking University Health Science Centre, Beijing, China; 4 Department of Neurology, Peking University First Hospital, Beijing, China; 5 Beijing Society of Rare Diseases, Beijing, China; 6 Department of Biostatistics, Peking University First Hospital, Beijing, China; 7 Peking University Clinical Research Institute, Beijing, China; 8 Department of Hospital Administration of Peking University Health Science Centre, Peking University, Beijing, China

**Keywords:** Rare diseases, Hospitalization, Database

## Abstract

**Background:**

There are many public health issues to resolve regarding rare diseases, including a lack of data from large-scale studies. The objective of this study was to explore fundamental data for a list of rare diseases in China, based on a hospitalization summary reports (HSRs) database. The Target Rare Diseases List (TRDL) 2017 was generated using an expert consensus method in which experts listed diseases according to research priorities. Using codes of the 10th revision of the International Statistical Classification of Diseases and Related Health Problems (ICD-10) and key search terms of rare diseases in English and Chinese, data were obtained from HSRs of 96 hospitals, covering a population of over 15 million in China from 2014 to 2015. We extracted and analyzed information on demographics, hospitalizations, and readmissions.

**Results:**

A total 281 rare diseases were included in the TRDL 2017. Altogether, 106,746 hospitalizations for a rare disease were captured from 1 January 2014 to 31 December 2015, accounting for 0.69% of inpatients during the same period. The top 10 rare diseases with most cases on the TRDL 2017 were thalassemia, idiopathic pulmonary arterial hypertension, pulmonary Langerhans cell histiocytosis, moyamoya disease, motor neuron disease, idiopathic pulmonary fibrosis, systemic sclerosis, hepatolenticular degeneration, coarctation of the aorta, and transposition of the great arteries. Among the 24 cities in the database, the five cities with the most types of the rare disease were Beijing, Changsha, Guangzhou, Shanghai, and Chengdu, with 191, 162, 143, 141, and 133 types, respectively. The five cities with most cases of the 281 rare diseases were Beijing, Guangzhou, Shanghai, Nanning, and Chengdu. The age distribution of rare diseases was 52% for the age group 25–64 years, and 27% of cases in the age group of 0–14 years were among children. The 10 highest readmission rates ranged from 35 to 65%.

**Conclusions:**

This study provided the TRDL 2017 and descriptive analysis of 281 rare diseases in a hospitalized population. Our study reveals important fundamental information that will be useful in national policy making and legislation; registry implementation; and diagnosis, treatment, and prevention of rare diseases in China.

**Electronic supplementary material:**

The online version of this article (10.1186/s13023-019-1137-y) contains supplementary material, which is available to authorized users.

## Background

The term rare diseases, also known as orphan diseases, refers to diseases with low prevalence, which do not yet have a universal definition [1]. According to the Activity Report of Orphanet in 2016, it was estimated that there are over 6900 rare diseases in the world [2].

With greater attention being directed to rare diseases worldwide, there has been an increasing number of studies of rare diseases and new drug development, with corresponding policies established in different countries and regions [3–6]. In recent years, more studies have been conducted on rare diseases globally, including clinical trials with numerous high-quality publications. There has also been increasing public awareness of rare diseases in China in recent years [7, 8]. However, epidemiological data for China are still lacking as there have been very few nationwide studies in the country [8]. The absence of such information makes it difficult to promote public awareness, facilitate health policy making and implementation, and provide medical resources.

Population-based research on rare diseases is arduous due to low disease prevalence and the high cost of such studies [9]. Moreover, many patients with rare diseases receive insufficient medical care. High costs for information acquisition adds to the difficulty as the information of such patients is usually unmeasurable and inaccessible. It is time-consuming, labor-intensive, and costly to perform population-based studies in China, with its population of over 1.3 billion.

The hospitalization summary reports (HSRs) database is a national mandatory patient-level database of hospitalized populations, under the management of the National Health Commission of the People’s Republic of China. The HSRs database contain medical record information, according to codes of the 10th Revision of the International Classification of Diseases (ICD-10).

A great many rare diseases have been identified worldwide. However, many of these diseases have various names in Chinese language, and others lack an appropriate ICD-10 code, which makes it difficult to perform surveys or studies. In addition, it is difficult to obtain firsthand data for as many as 6900 rare diseases based on hospitalized patients, given the inaccurate names used for these diseases. In consideration of the difficulty in clarifying and correcting the Chinese names for rare diseases, this study was conducted based on a Target Rare Diseases List (TRDL) in China, created using expert consensus.

The main objective of this study was to develop the TRDL 2017 using an expert consensus method and to explore the fundamental data of rare diseases on the TRDL 2017 based on the HSRs database in China during 2014 to 2015, with a particular focus on the number of hospitalizations, city and age distribution, and readmission rate.

## Methods

### Development of the TRDL 2017

In the first step of creating the TRDL 2017, rare disease names were summarized according to four available lists of rare disease names in China. These four sources included recommendations for the rare disease name list made by experts of the National Health Commission of the People’s Republic of China that was meant to improve ICD coding and funding reimbursement of therapies, experts from the Beijing Society of Rare Diseases for epidemiologic surveillance, the book entitled *Treatable Rare Diseases* [10] for scientific popularization of meteorites, and a national study on a partial registry of rare diseases (the National Key Research and Development Program of China clinical cohort study of rare diseases (2016YFC0901500)) that was a national fund project for rare disease research.

In the next step, after removing duplicate names, we obtained a primary list with 344 rare diseases by summarizing and proofreading disease names from the four list sources mentioned above.

**Fig. 1:**
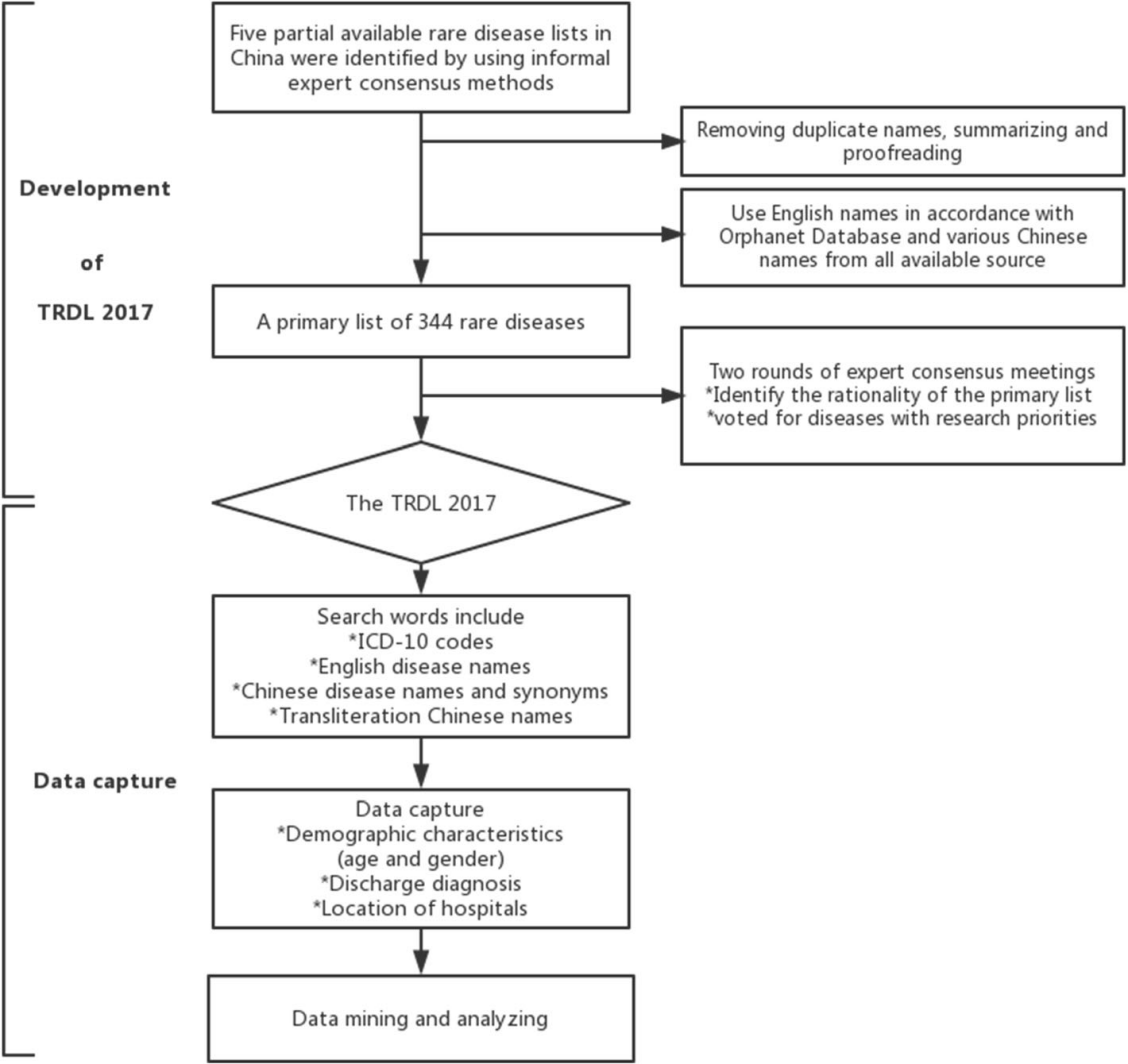
Flowchart of TRDL 2017 development and data capture. TRDL, Target Rare Diseases List

In the third step, two expert consensus meetings were held. In the first meeting, 18 experts from across China were invited to individually explain their rationale for the primary list as well as the methodology involved, via public discussions. The professional fields of the 18 experts included pediatrics, neurology, respiratory medicine, ophthalmology, genetics, pharmacy, epidemiology, statistics, mathematics, and information science. In the second consensus meeting, another group of 21 experts first held public discussions and then voted by anonymous ballot for those diseases with the highest research priorities. The final TRDL 2017 was formulated based on the results of this expert consensus. The experts who took part in the two expert consensus meetings were all senior experts on relevant rare diseases nationwide. The flowchart of development of the TRDL 2017 is shown in Fig. 1.

### Study population and data sources

Data were extracted from the database of hospitalization summary reports (HSRs). This is a patient-level national database of hospitalized populations. The selected hospitals submit HSRs to the HSR system annually, in accordance with requirements of the National Health Commission of the People’s Republic of China [11–14]. The HSR system includes data integration, data storage and management, data analysis and mining, and results display. Each layer guarantees data safety and quality control [15].

The database covers 96 tertiary hospitals in 25 provinces across China. All 96 hospitals are university affiliated hospitals or provincial hospitals. For each patient in the HSRs database, clinical information includes demographic characteristics (age, sex), discharge diagnosis, location of the hospital, and corresponding ICD-10 codes.

Target rare diseases in the TRDL 2017 were identified according to discharge ICD-10 codes. The flowchart of data capture is shown in Fig. 1.

### Data analysis

Demographic information about the study population and their admissions to tertiary hospitals during 2014 to 2015 in China, including the number of hospitalizations, male to female ratio, city distribution, age distribution and readmission rate.

Rare diseases were analyzed by their ICD-10 codes. Correctly identifying disease names in the HSRs database is complex as the database contains English names, names in both English and Chinese, transliteration of Chinese names, and synonyms. To minimize possible inaccuracy of disease coding and names, both ICD-10 codes and key search terms (in English and Chinese) of rare diseases were used for data capture. In addition, a few rare diseases lacking ICD-10 codes were identified using search terms (in English and Chinese). The total number of hospitalizations, total cases of rare diseases on TRDL 2017, the top ten rare diseases with most cases and the rare diseases with no more than one case were calculated.

Patients’ information on the residential province of patients could not be obtained; therefore, hospital locations were used for city distribution. The five cities with the most types and the five cities with the most cases of rare disease listed on the TRDL 2017 were calculated.

Patients’ age at admission was used for analysis of age distribution. The age group included 0–4 years, 5–14 years, 15–24 years, 25–34 years, 35–44 years, 45–54 years, 55–64 years, 65–74 years, 75–84 years, 85~ years. The number of these ten age groups were calculated.

Hospitalizations of patients in the same hospital could be identified, but not in different hopitals due to the deidentification and encryption of patient data. So readmission in this study refers to rehospitalization in the same hospital.

Continuous data were described using mean and standard deviation; and categorical variables were presented as frequency and proportion. All statistical analyses were performed using R (version 3.5.1).

## Results

A total of 281 rare diseases from the four source lists were included on the TRDL 2017 (Additional file 1). Altogether, we captured data of 106,746 hospitalizations for one of these 281 rare diseases, in the 96 included hospitals between 1 January 2014 to 31 December 2015; these cases were included in the current study, with 50,555 and 56,191 cases in 2014 and 2015, respectively. The overall number of hospitalized patients in the HSRs database was 15,458,065; there were 7,429,813 and 8,028,252 cases in 2014 and 2015, respectively. Patients hospitalized with any of the 281 rare diseases during 2014–2015 accounted for 0.69% of inpatients during the same period, with 0.68 and 0.70% in 2014 and 2015, respectively.

**Fig. 2:**
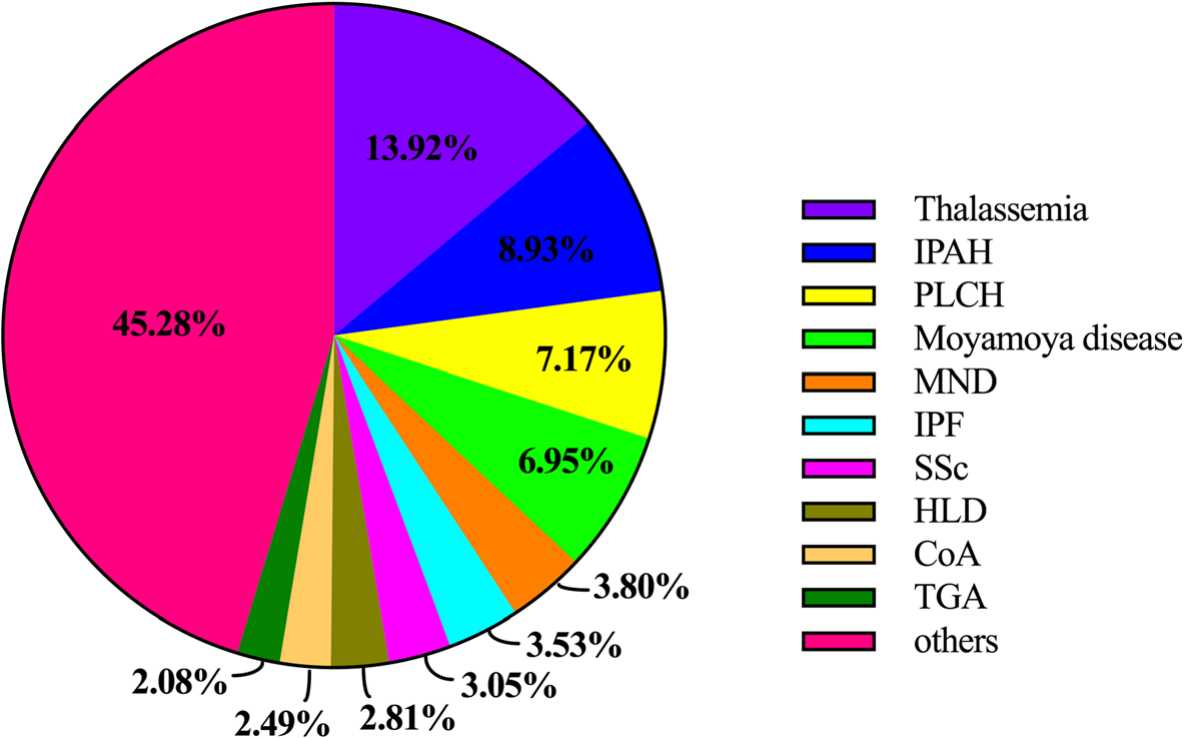
The percentage of the top 10 rare diseases with most cases and other diseases on the Target Rare Diseases List 2017

**Fig. 3:**
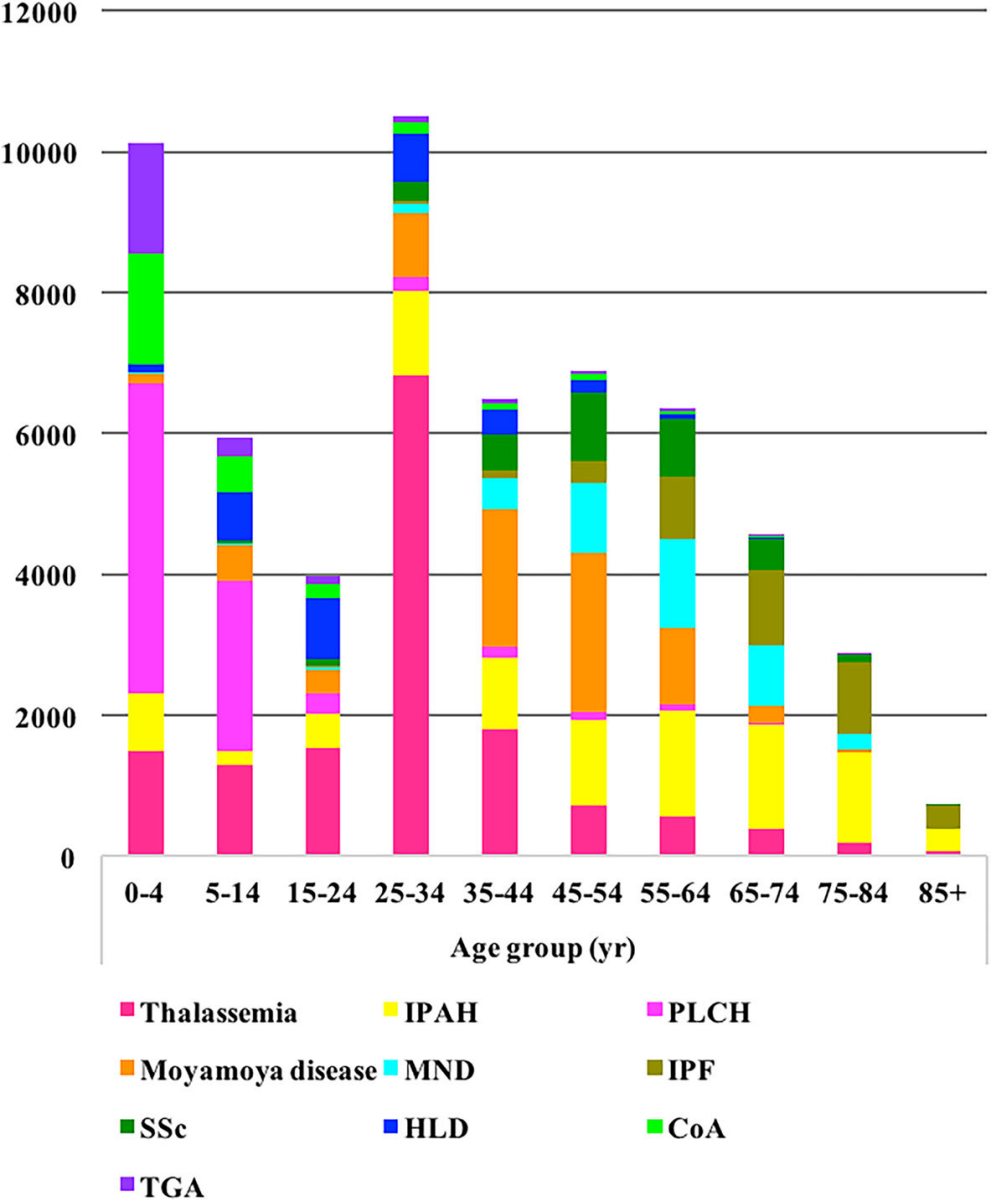
Age distribution of the top 10 rare diseases with most cases on the Target Rare Diseases List 2017. CoA: coarctation of the aorta; HLD: hepatolenticular degeneration; IPAH: idiopathic pulmonary arterial hypertension; IPF: idiopathic pulmonary fibrosis; MND: motor neuron disease; PLCH: pulmonary Langerhans cell histiocytosis; SSc: systemic sclerosis; TGA: transposition of the great arteries

The top 10 rare diseases with most cases accounted for 54.7% (*N* = 58,415/106,746) of the 281 rare diseases listed on the TRDL 2017, and 0.38% (*N* = 58,415/15,458,065) of hospitalized inpatients during 2014–2015. The general characteristics and number of cases for each of the 10 most frequent rare diseases are summarized in Table 1 and the percentage of the top 10 rare diseases with most cases and other diseases are shown in Fig. 2. The age distribution of cases among the 10 most frequent rare diseases are shown in Fig. 3.

**Table 1:** General characteristics of the top 10 rare diseases with most cases on the Target Rare Diseases List 2017

Disease	Total No.	2014			2015		
		N	Male N (%)	Age (yr, mean ± SD)	N	Male N (%)	Age (yr, mean ± SD)
Thalassemia	14,855	6782	1761 (25.97%)	28.5 ± 17.0	8073	1836 (22.74%)	29.4 ± 16.5
IPAH	9536	4661	1837 (39.41%)	46.7 ± 24.7	4875	1934 (39.67%)	51.6 ± 23.9
PLCH	7657	3302	2114 (64.02%)	7.1 ± 10.5	4355	2799 (64.27%)	7.7 ± 11.5
Moyamoya disease	7419	3441	1642 (47.72%)	41.0 ± 15.3	3978	1834 (46.10%)	42.3 ± 15.1
MND	4057	2030	1331 (65.57%)	55.8 ± 13.9	2027	1347 (66.45%)	56.7 ± 12.8
IPF	3764	1470	1001 (68.10%)	67.3 ± 12.8	2294	1579 (68.83%)	69.0 ± 13.1
SSc	3252	1522	286 (18.80%)	51.5 ± 13.8	1730	335 (19.36%)	51.2 ± 14.2
HLD	3000	1433	773 (53.94%)	23.9 ± 13.3	1567	887 (56.60%)	24.9 ± 14.2
CoA	2654	1269	865 (68.16%)	9.2 ± 14.8	1385	893 (64.48%)	10.7 ± 16.4
TGA	2221	1082	707 (65.34%)	6.6 ± 13.1	1139	757 (66.46%)	7.8 ± 14.4

*CoA* coarctation of the aorta, *HLD* hepatolenticular degeneration, *IPAH* idiopathic pulmonary arterial hypertension, *IPF* idiopathic pulmonary fibrosis, *MND* motor neuron disease, *PLCH* pulmonary Langerhans cell histiocytosis, *SSc* systemic sclerosis, *TGA* transposition of the great arteries

Among the 281 rare diseases, 77 had no more than 1 case each. The total cases for these 77 diseases accounted for 0.01% (15/106,746) of cases of the 281 rare diseases and only 0.0001% (*N* = 15/15,458,065) of the total inpatients during the study period. The number of hospitalizations for each rare disease on the TRDL 2017 and its comparison with the official “First Rare Diseases Catalogue” are shown in Additional file 2.

**Fig. 4:**
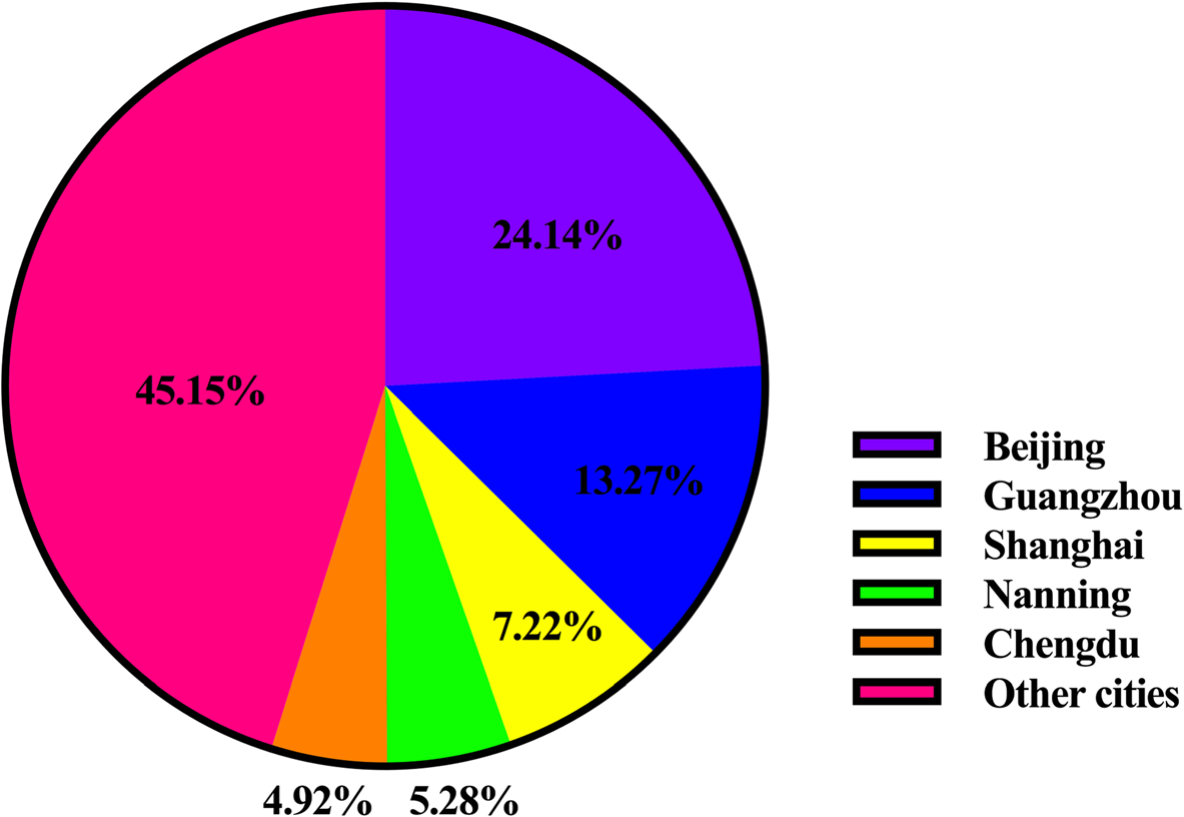
City distribution of cases for the 281 rare disease on the Target Rare Diseases List 2017 (during 2014–2015)

Among the 24 cities in the database, the five cities with the most types of rare disease listed on the TRDL 2017 were Beijing, Changsha, Guangzhou, Shanghai, and Chengdu, with 191, 162, 143, 141, and 133 types, respectively. The five cities with the most cases of the 281 rare diseases were Beijing, Guangzhou, Shanghai, Nanning, and Chengdu. The city distribution is shown in Fig. 4.

**Fig. 5:**
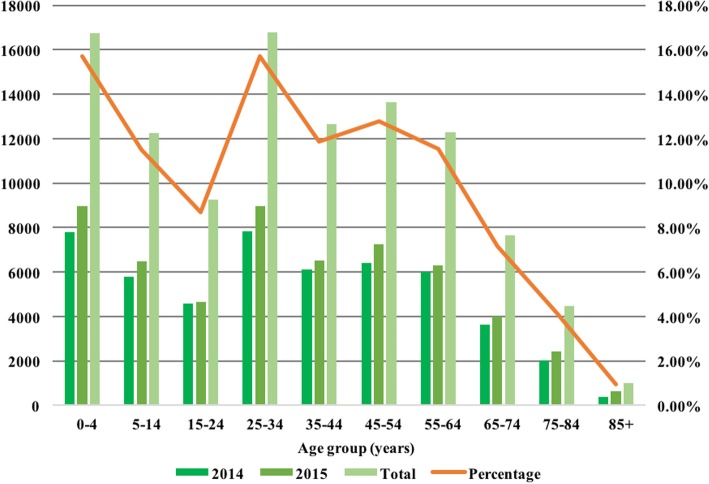
Age distribution of cases for the 281 rare disease on the Target Rare Diseases List 2017 (during 2014–2015)

The total number of rare disease cases in 2014 and 2015 was 106,746, of which 50.4% occurred in male patients (*N* = 53,852) and 49.6% in female patients (*N* = 52,894). The age stratification and percentages of cases are illustrated in Fig. 5.

Among the 281 rare diseases on the TRDL 2017, the 10 diseases with the highest readmission rates in 2014 and 2015 are shown in Table 2.

**Table 2:** The 10 rare diseases on the Target Rare Diseases List 2017 with the highest rates of readmission (2014–2015)

Name of disease	No. of readmission	No. of hospitalizations	Readmission rate^a^
PLCH	4968	7657	64.88%
Arginase deficiency	9	14	64.29%
BS	22	40	55.00%
Osteopetrosis	115	211	54.50%
XHIM	1	2	50.00%
XLA	44	109	40.37%
WAS	2	5	40.00%
HUS	205	532	38.53%
CD	111	301	36.88%
XP	19	54	35.19%

*BS* Blau syndrome, *CD* Castleman disease, *HUS* hemolytic uremic syndrome, *PLCH* pulmonary Langerhans cell histiocytosis, *WAS* Wiskott–Aldrich syndrome, *XHIM* X-linked hyper IgM syndrome, *XLA* X-linked agammaglobulinemia, *XP* xeroderma pigmentosum

^a^Proportion with rehospitalization in the same hospital

## Discussion

At present, this is the first nationwide study of rare diseases among hospitalized populations in China based on a large, high-quality dataset of HSRs. All hospitals covered are tertiary hospitals where physicians are highly qualified in the diagnosis and treatment of rare diseases, which renders the HSRs database of high quality and suitable for the study of rare diseases.

Our study showed that the 10 most frequently occurring rare diseases among those on the generated TRDL 2017, ranged from 2221 to 14,855 cases. Of the 281 rare diseases, 77 had no more than one case registered in the database, which indicated a large gap in the number of patients with different rare diseases. According to published articles for each of these 77 rare diseases in China, the number of cases might be underestimated in this study. For instance, between 2014 to 2015, the following diseases had more than one reported case in China: isovaleric acidemia [16–18], ornithine transcarbamylase deficiency [19, 20], glutaric acidemia type I [21, 22], leukoencephalopathy with calcifications and cysts [23, 24], Alexander disease [25–28], myoclonic epilepsy with ragged red fibers [29, 30], and Pelizaeus–Merzbacher disease [31].

In this study, the city distribution of patients with rare diseases was concentrated in Beijing, Shanghai, Guangzhou, and Chengdu, which may indicate that hospitals in these four cities are more capable of diagnosing and treating rare diseases. However, people in China crowd together in large cities, particularly in the abovementioned cities; therefore, the number of hospitalizations for rare diseases can be expected to be much higher in these four cities than in other cities.

There was no difference in terms of the proportions of cases of the 281 rare diseases among hospitalizations between 2014 (0.68%) and 2015 (0.70%), which might indicate that the diagnosis and treatment status of rare diseases is relatively stable in China.

The age distribution showed that hospitalizations for the rare diseases on the TRDL 2017 in the age group 25–64 years, known as working age, accounted for 51.87% of cases, which might reflect a family, social, and economic burden for patients with rare diseases. The number of cases of the 281 rare diseases among children aged 0–14 accounted for 27.19% of cases, which clearly shows that children represent a high percentage of patients with these rare diseases. Of the total 281 rare diseases, the 10 with the highest readmission rates had rehospitalization rates ranging from 35.19 to 64.88%. These readmission data may be useful in analyses of the financial burden of rare diseases, although health care costs cannot currently be obtained from the HSRs database.

### Strengths

The present study is the first national survey of rare diseases in China and included the largest study population to date. Second, the process from development of the TRDL 2017 to data capture and analysis was rigorous. Third, based on a systematic methodology, we established the TRDL 2017 is a feasible way, and the list can be continuously and quickly updated for further study. Finally, our study will contribute to updating the World Health Organization nomenclature for rare diseases in China in that we standardized the names of 281 rare diseases between English and Chinese language.

### Limitations

Although this hospitalized population-based study could describe the fundamental data of a sizable group of rare diseases, underreporting of rare disease cases is inevitable for three reasons. First, the HSR data are limited to hospitalized patients. Second, all hospitals involved in this study are all tertiary hospitals, but not all tertiary hospitals in China were included in the database. Third, tertiary hospitals in China also provide primary, secondary and tertiary care and have the exposure to nationwide patient population due to the lack of hierarchical referral system, which was different from the tertiary hospitals of western medical system, so prevalence in each city could not be obtained. Fourth, by cross-matching our TRDL list to Orphanet nomenclature of RD, we found that most diseases in our list are single diseases, and some are groups of diseases, which may lack precise ICD-10 codes so could not be extracted from the database. Fifth, mismatching of rare disease nomenclature may have resulted in the exclusion of some patients. Sixth, the statistical results of the research data are biased caused by the fact that the current registration information of inpatients in different hospitals in China cannot be shared so the hospitalization number of same patient with rare disease admitted to different hospitals cannot be offset. For example, rehospitalization rate was underestimated as the rehospitalized cases only represent inpatient cases in the same hospital. Consequently, individual-level data could not be acquired in the present study. Seventh, residential place of the hospitalized patients is not an essential parameter in the database. Therefore, the distribution of the patient population by city are unclear. Lastly, the final selection of the 281 diseases on the TRDL 2017 was determined by anonymous ballot as those diseases with considered to have the highest research priority, which makes this list very different from those of other publications focusing on disease frequency. However, our results still fill a gap in the data for rare diseases in China. It is the largest and most complete dataset with important reference value.

## Conclusions

This study provided a list, the TRDL 2017, and a descriptive analysis of rare diseases in hospitalized populations in China. Our study provides important and fundamental data for policy making and legislation; registry implementation; and the diagnosis, treatment, and prevention of rare diseases in China.

## Additional files

Additional file 1: The four lists of TRDL 2017. (XLSX 42 kb)

Additional file 2: The number of hospitalizations with each rare disease in TRDL 2017. (XLSX 275 kb)

## Data Availability

All data generated or analyzed during the study are included in this published article and the additional files.

## Abbreviations

BS: Blau syndrome
CD: Castleman disease
CoA: Coarctation of the aorta
HLD: Hepatolenticular degeneration
HSRs: hospitalization summary reports
HUS: Hemolytic uremic syndrome
ICD-10: the 10th Revision of the International Classification of Diseases
IPAH: Idiopathic pulmonary arterial hypertension
IPF: Idiopathic pulmonary fibrosis
MND: Motor neuron disease
No.: Number
PLCH: Pulmonary Langerhans’cell histiocytosis
PNH: Paroxysmal nocturnal hemoglobinuria
SSc: Systemic sclerosis
TGA: Transposition of great artery
TRDL: Target Rare Diseases List
WAS: Wiskott-Aldrich syndrome
XHIM: X-linked hyper IgM syndrome
XLA: X-linked agammaglobulinemia
XP: Xeroderma pigmentosum
